# Orientation selectivity in a model of primary visual cortex with and without orientation map

**DOI:** 10.1186/1471-2202-16-S1-P160

**Published:** 2015-12-18

**Authors:** Soledad Gonzalo Cogno, Germán Mato

**Affiliations:** 1 Statistical and Interdisciplinary Physics Group, Instituto Balseiro and Centro Atómico Bariloche, Bariloche, 8400, Argentina

## 

Since its discovery by Hubel and Wiesel in 1959, orientation selectivity has been observed in every mammal for which the neuronal response selectivity of primary visual cortex (V1) has been examined. In some animals, like cats and monkeys, anatomically close V1 neurons have similar preferred orientations, giving rise to maps of orientation preferences. However, sharp selectivity is also observed in mice, squirrels and rats, whose V1 has no orientation map. This means that neurons with different preferred orientations are intermixed. This second scenario is called salt-and-pepper organization and leads to question the structural organization of the intracortical connections.

With this study, we intend to analyze the differences between both scenarios focusing on selectivity properties, and to clarify the effect of plasticity on orientation selectivity. We study a computational model of layer 2/3 and a reduced one-dimensional model of orientation selective neurons, both in the balanced state. We also analyze a plasticity mechanism that involves spike-timing dependent plasticity (STDP) [1]. This rule implies that if the postsynaptic spike comes after the presynaptic spike, the connection becomes stronger. For the reverse order it becomes weaker. Inhibitory synapses are taken as non-plastic. The selectivity is quantified using the Orientation Selectivity Index (OSI) of the activity profile.

According to the theory of balanced networks [2] applied to spatially structured networks [3], we find that for a given profile of the input, selectivity of the cortical activity is determined by the ratio between the first Fourier component of the connectivity and its mean value. As a consequence, for large connectivity salt-and-pepper structures are more selective than systems with orientation maps: our simulations indicate that the mean OSI for salt-and-pepper is 0.57, while it is 0.27 for orientation map. Moreover, for systems with orientation maps selectivity could be increased by taking connection probabilities that are broader.

Regarding plasticity, we find that under certain conditions STDP can indeed improve selectivity but it works in a somehow unexpected way, by decreasing the modulated part of the intracortical connectivity with respect to the non-modulated part. We find this conclusion to be valid both for systems with salt-and-pepper organization and with orientation maps. This can be understood in terms of the relative change between the background connectivity and the functionally modulated part.
